# Controlled Focused Ion Beam Milling of Composite Solid State Nanopore Arrays for Molecule Sensing

**DOI:** 10.3390/mi10110774

**Published:** 2019-11-13

**Authors:** Péter Fürjes

**Affiliations:** Microsystems Lab, Inst. of Technical Physics and Materials Science, Centre for Energy Research, Budapest H-1121, Hungary; furjes@mfa.kfki.hu

**Keywords:** nanofluidics, nanofabrication, focused ion beam milling, composite/multi-layer solid state nanopore array, molecule detection

## Abstract

Various nanoscale fabrication techniques are elaborated to form artificial nanoporous/nanochannel membranes to be applied for biosensing: one of the most prevalent is the micro-electromechanical systems (MEMS) compatible focused ion beam (FIB) milling. This technique can be easily adopted in micro- and nanomachining process sequences to develop composite multi-pore structures, although its precision and reproducibility are key points in the case of these thick multi-layered membranes. This work is to demonstrate a comprehensive characterisation of FIB milling to improve the reliability of the fabrication of solid state nanopore arrays with precisely predetermined pore geometries for a targeted molecule type to be recognised. The statistical geometric features of the fabricated nanopores were recorded as the function of the process parameters, and the resulting geometries were analysed in detail by high resolution scanning electron microscope (SEM), transmission electron microscope (TEM) and ion scanning microscopy. Continuous function of the pore diameter evolution rate was derived from the experimental results in the case of different material structures, and compared to former dissentient estimations. The additional metal layer was deposited onto the backside of the membrane and grounded during the ion milling to prevent the electrical charging of dielectric layers. The study proved that the conformity of the pore geometry and the reliability of their fabrication could be improved significantly. The applicability of the developed nanopore arrays for molecule detection was also considered by characterising the pore diameter dependent sensitivity of the membrane impedance modulation based measurement method.

## 1. Introduction

Compared to the macroscopic case, self-confined nanoscale structures exhibit significantly different chemical and transport behaviours. The understanding of the involved chemical and physical phenomena opens the way to explore the “world of nanofluidics” [1], and to develop brand new sensing principles on the molecular or sub-molecular level.

### 1.1. Transport in Nanoscale

Regarding biosensor development, the application of microfluidic systems for liquid sample management, including particle separation, reagent mixing, transport, etc., is essential [2]. By decreasing the characteristic dimensions of the fluidic transport systems, significantly different governing physical phenomena can be experienced, which become dominant in the nanofluidic systems by stepping over the critical size of <100 nm. Approaching the characteristic lengths of electrostatic, intermolecular or hydrodynamic interactions, interfacial phenomena increasingly dominate the physical behaviour of the system [3].

The special effects revealed in the nanoconfinements are [1]:

- Permselectivity induces asymmetric pressure or electric field driven molecule transport through the nanochannels or nanopores.
- Size dependent transpore permeation of molecules is caused by the “Born repulsion” evolved due to the electrical interaction between the overlapping electron clouds of translocating molecules and atoms of the pore surface.
- Reconfiguration of macromolecules can also be experienced (in the case of deoxyribonucleic acid (DNA) chains) due to the entropic barrier conditions.

Considering dimension-dependent transport in the nanochannels, the pressure driven flow rates scale inversely to the channel cross section, contrary to the electroosmotic and electrophoretic flows.

### 1.2. Applications of Nanofluidic Systems

The most relevant envisioned applications of nanofluidic systems range from molecule separation (both concentration and accumulation), molecule detection, to drug release and delivery, but 3rd generation DNA sequencing may also be one of the important fields utilising these structures. Concerning the widespread applications of nanofluidic structures, there are also several challenges to master. The main obstacles are [1,4]:

- Development of robust and engineered fabrication techniques for reproducible nanostructure formation.
- Clarification the comprehensive theory and construction predictive modelling of nanoscale transport phenomena.
- Precise control and in-situ monitoring of physical or chemical parameters (e.g., transport properties or local surface modification) inside the nanopores.

On the other hand, focusing on molecule recognition or concentration detection, the following strengths of nanofluidic structures should be underlined: the possibility of label-free, specific molecule (or single molecule) detection with extreme sensitivity and the transport modulation-based built-in amplification by nanoconfinement in nanopores, as discussed by Gyurcsányi [4].

**Figure 1:**
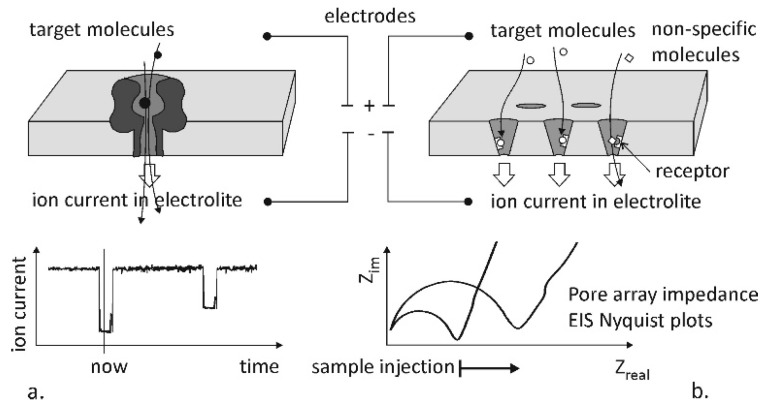
Schematic representation of the principle of stochastic molecule detection and counting (**a**) and the membrane impedance analysis approach (**b**).

Two main molecule sensing principles based on the ionic current modulation are visualized in Figure 1: the translocation or the capturing of molecules in nanochannels or nanopores [5]. The stochastic sensing by a single channel (Figure 1a) makes use of the monitoring of the ionic current through the nanopore or nanochannel. The current spikes are recorded and counted as single molecule translocation events. This principle regarding biomolecule recognition [6] was studied by Bayley and Cremer [7] and is applied in the devices of the Oxford Nanopore Technologies [8,9]. The applicability of the method was also demonstrated for counting and sizing viruses by monitoring the translocation effect in glass nanopipets [10]. The other approach is the analysis of the impedance of a membrane (Figure 1b) containing a nanopore array (multiple nanopores), which is mostly applied in the case of functionalised nanoconfinements systems, like the ones examined in the present paper, In these structures, Electrochemical impedance spectroscopy (EIS) is performed during the translocation and binding events [11,12,13]. The reliable biofunctionalisation of the specific areas of these nanostructures is quite challenging, therefore, the optimal material selection and structure are in the focus of the development of molecule sensing devices [14].

### 1.3. Nanopore Structures and Their Fabrication Techniques

Extremely sensitive label free detection method can be achieved by monitoring pore-through transport modulated by translocation or binding of the target molecules in nanopores. The sensitivity of these mainly electrochemical (bio)sensors are determined not only by the biofuncionalisation but affected by the pore geometry also: it must be aligned to the size and conformation of the target molecule. Moreover, the comparability and repeatability of the measurements assumes similar pore parameters in case of different devices. Accordingly, the practical applicability and potential commercialisation of these nanopore membrane based devices depend on the successful elaboration of controllable and reproducible pore fabrication techniques. Generally, three typical nanopore membrane structures can be defined regarding their membrane materials and nanopore formation methods [15,16,17]:

- Biological nanoporous layers are mainly based on pore-forming transmembrane proteins embedded in lipid bilayer membranes.
- Hybrid type nanopore membranes consist of similar biological pore-forming molecules integrated in synthetic layers.
- Artificial nanopore structures are fabricated by dedicated nanoprocessing methods in synthetic materials, polymer, glass, quartz or dielectric layers compatible with silicon technology (silicon-nitride, silicon-oxide, silicon-carbide, possibly Atomic Layer Deposited metal-oxides), or 2D materials (graphene, molybdenum-oxide, etc.).

The applicability of the biological nanopores or nanochannels for transport modulation based molecule recognition and counting was demonstrated extensively. Their main advantages are the self-controlled size and shape, and the favorable costs. It can be noticed that the limited geometrical and material variability, the uncertain integrability and the moderate resistivity of the biological structures against pH, ion concentration, temperature, mechanical stress, etc. however, restrict their reliable applicability in diagnostic systems [17]. A well-published sample for the biological and hybrid structures is the embedded alpha-haemolysin, which was demonstrated in different applications for molecule detection [18,19] or ultrafast DNA sequencing [15,16,20,21,22]. MspA (Mycobacterium smegmatis porin A) and phi29 connector proteins were also demonstrated in DNA sequencing [23]. Solid-state nanopores and their nanofabrication techniques in dielectric layers were demonstrated and assigned as the novel generation of artificial nanopore membranes by Golovchenko, Dekker and Edel’s group [17,24,25]. The flexible variability in shape, size and surface properties, and the robustness and integrability were emphasized as the main advantages of the solid state nanostructures [26,27,28,29]. The exploitation of these artificial nanopores for molecule sensing and DNA analysis was demonstrated in diverse biomedical applications [30,31,32,33].

Several high-tech solutions were described for more or less precise nanopore fabrication in the range from 2 nm to several tens of nanometres utilizing the capabilities of nanofabrication equipment as summarized in Table 1 [34,35]. The frequently applied membrane materials are silicon-nitride, silicon-oxide, silicon-carbide or atomic layer deposited (ALD) aluminium-oxide, titanium-oxide, hafnium-oxide, i.e., mainly MEMS compatible dielectrics, or 2D materials, as graphene and molybdenum-oxide [15,16,36]. Accordingly, the solid state structures provide the significant advantage of controllable shape and size, the excellent mechanical, thermal and chemical stability and integrability in microfluidic systems [17]. Note, however, that the fabrication can be costly and time-consuming in the case of chip-scale processing. The most extensively applied techniques are:

- etching after e-beam lithography based patterning [17,34,35,37],
- ion sculpturing or Focused Ion Beam (Ga^+^, Ar^+^, He^2+^, Xe^+^, etc.) milling [17,24,25,31,34,35,36],
- high intensity electron beam drilling or ablation in TEM [25,34,35,36],
- Nanoimprint Lithography (NIL) [34],
- micromachining (MEMS based) techniques as nanochannel formation and subsequent sealing by bonding [34].

Attaining nanometer scale pore sizes is doubtless challenging, and to shrink the previously formed pores additional deposition techniques are required, like electrochemical or chemical vapour and atomic layer deposition [38,39] or local Ion and Electron Beam Assisted Deposition (EBAD or IBAD) [40,41]. The final pore size and geometry can also be modified by a high intensity wide-field electron illumination reflowing and reshaping the pore forming dielectric material due to the surface tension [17]. Controlled dielectric breakdown of the membrane material was also reported to form single nanopores of extremely small diameter. [42,43] Single and multiple nanopores were formed in precisely controlled positions by initiation the dielectric breakdown locally using a conductive AFM (Atomic Force Microscopy) tip as electrode. [44] To fabricate multiple nanopores with larger diameter the controlled breakdown was combined by local optical heating of the dielectric membrane by visible laser light. [45,46] Focused laser beam induced optical etching was also applied to fabricate nanopores in SiN_x_ membrane. [47] The evolving 10-20nm pore size is comparable to the achievable parameters of FIB milling.

The key to the commercialization of nanopore based biosensors or Lab-on-a-Chip devices is the development of adequate nanoprocessing techniques ensuring fast, high-throughput, controllable and reproducible formation of the application specific nanopore geometries in differently structured solid state membranes [31]. Adequate material composition of the membrane can moderate the residual mechanical stress of the suspended structures [48]. Moreover, to fulfil the crucial requirement of easy and area or material selective biofunctionalisation of the inner pore surfaces, the application and planned shaping of multi-layered (composite) membranes have elevated importance [35]. These multi-layered solid state nanopore arrays integrable electrically and fluidically could be core elements of extremely sensitive label-free, multi-parametric molecule recognition.

The direct milling of complex composite membrane structures by focused electron or ion beams could be an obvious solution to fabricate nanopore arrays with the proposed geometries. Note, that in the case of thicker or composite membranes the precise fine-tuning of the pore size can be a critical point of the reliable and reproducible fabrication due to the beam-shape (defocusing), redeposition and charging of the dielectric membranes [49]. Accordingly, the comprehensive study of the pore formation process is crucial to achieve adequate precision and reproducibility in the case of these complex layer structures.

## 2. Materials and Methods

### 2.1. Fabrication of Nanopore Arrays by Focused Ion Beam (FIB)

Solid state nanopores were prepared by computer controlled Focused Ion Beam (FIB) milling using accelerated Ga^+^ ions with different milling currents and doses, in order to achieve various pore geometries in multi-layered membranes made of different dielectrics. The adopted structural and material combination has to be compatible to the proposed biofunctionalisation strategies (see Table 2).

**Figure 2:**
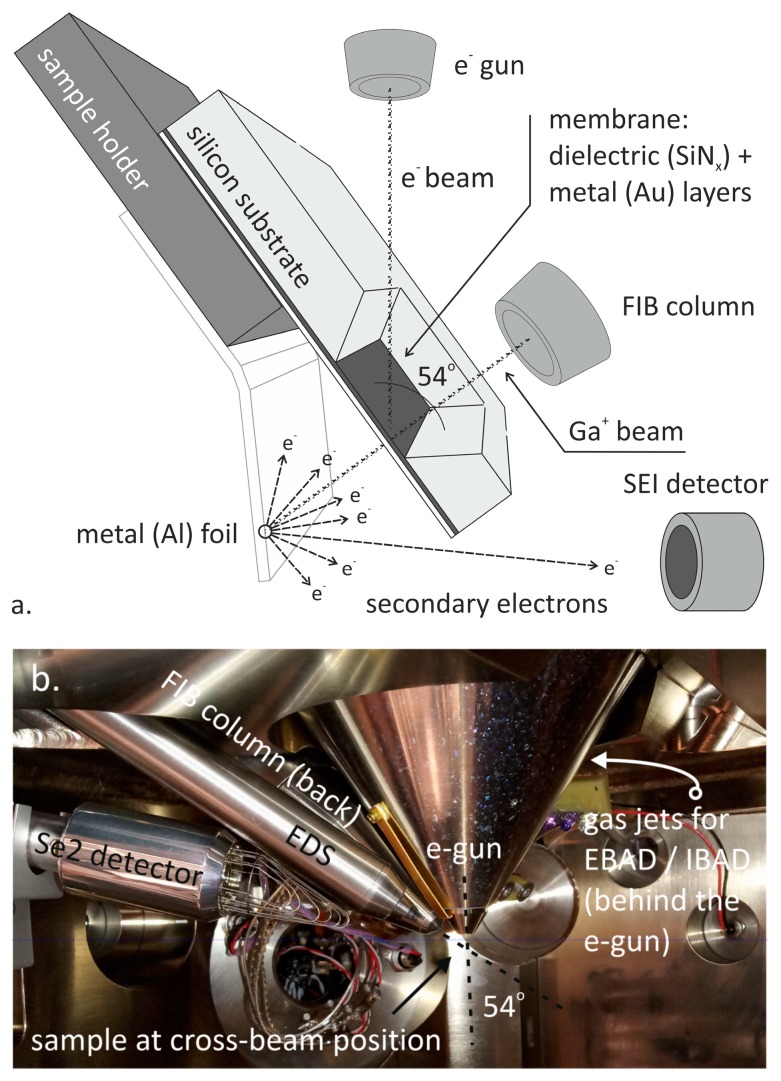
Schematic representation of the sample geometry for nanopore fabrication and analysis (**a**) and the real cross-beam setup in Zeiss LEO 1540 XB FESEM/FIB nanoprocessing system adjusted for FIB milling (**b**).

The structures were fabricated by the combination of micro- and nanomachining technologies. The dielectric membrane was a 300 nm thick non-stoichiometric silicon-nitride film prepared by Low Pressure Chemical Vapour Deposition (Tempress LPCVD, Tempress, Vaassen, The Netherlands) using ammonia (NH_3_)/dichlorosilane (H_2_Cl_2_Si) mixture (with 1:8 flow rate ratio) at 180 mTorr backpressure and temperature of 830 °C. Non-stoichiometric silicon-nitride films have moderate mechanical stress ensuring a adequate mechanical stability of the suspended membrane. The covering gold layer (150 nm) was deposited in the same cycle by vacuum evaporation atop of 5 nm of titanium adhesion layer in an AJA Orion high vacuum system. The dielectric membrane was released by wet alkaline (KOH at 72 °C) or Deep Reactive Ion Etching (DRIE) etching using the „Bosch-process” (Oxford Plasmalab 100, Oxford Instruments Plasma Technology, Bristol, UK) from the backside, and then the nanopores were drilled by employing a program controlled Ga^+^ FIB drilling using the feature milling option of the Zeiss LEO 1540XB (Carl Zeiss Microscopy GmbH, Jena, Germany) nanofabrication system. The schematic representation of the pore fabrication process and the sample placement in cross-beam position under the FIB/SEM guns is shown in Figure 2. The ion beam was aligned, focused and set in cross-beam position outside of the membrane, close to the frame, to prevent its perforation. The milling current and time were varied to achieve the proposed pore geometry with 30 keV Ga^+^ ion energy.

### 2.2. Characterisation of Nanopore Geometries

**Figure 3:**
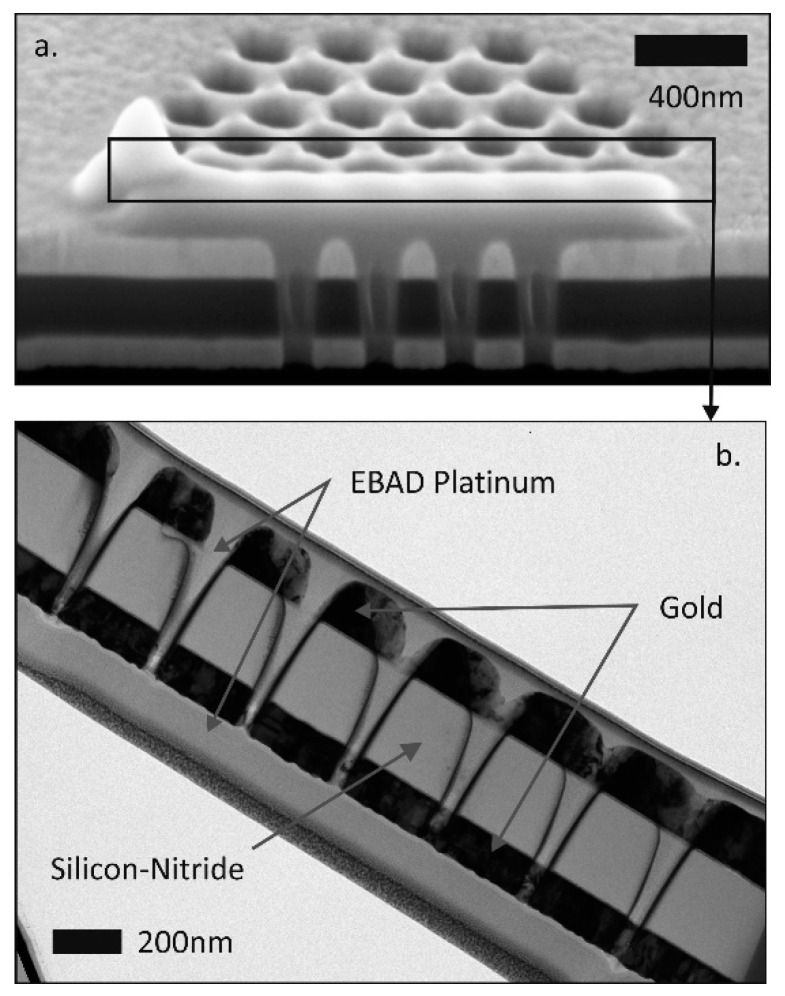
Cross-sectional scanning electron microscope (SEM) (**a**) and transmission electron microscope (TEM) (**b**) view of the nanopore array revealed by FIB milling of an Au/SiNx/Au membrane.

The pore geometries were characterised by SEM, TEM and ion scanning microscopy after cross sectioning by FIB (Figure 3). The nanopores were filled with Platinum applying local Electron Beam Assisted Deposition (EBAD) to achieve the sufficient mechanical stability of the membrane during preparation of the TEM lamella and during TEM examination. Trimethyl(methylcyclopentadienyl)platinum(IV) (C_5_H_4_CH_3_Pt(CH_3_)_3_) was injected as precursor from the Gas Injection System (GIS). Resulting pore diameters and the development of the pore shape during ion milling (aspect ratio, wall angles) had to be deduced.

**Figure 4:**
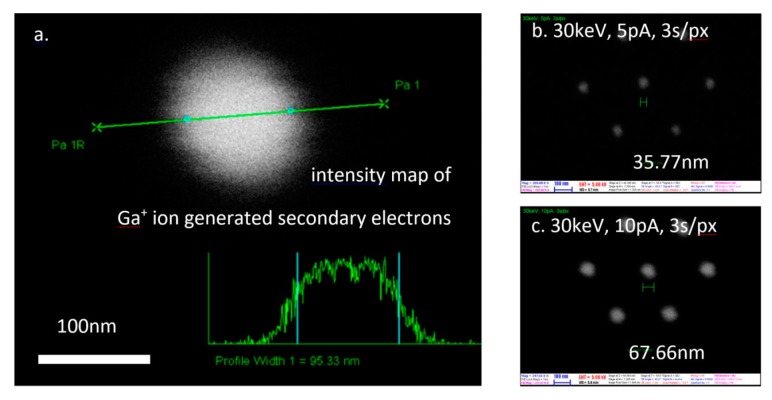
Pore diameter analysis by scanning ion microscopy (**a**) based on the detection secondary electrons generated by the ions translocated through the nanopore (see schematic in Figure 2). High number of fabricated nanopore were characterized for statistical results (**b**,**c**).

The obtained main structural parameters of the nanopores (e.g., pore diameter, wall angle) were recorded and analysed as a function of different manufacturing conditions (e.g., Ga^+^ ion current, milling time or ion dose). The pore diameters were in-situ analysed using scanning ion- and electron microscopy as the Ga^+^ ions and electrons passing through the pores precisely map the fabricated pores, as plotted in Figure 4. The translocation of the ions or electrons were determined by the detection of secondary electrons generated in an Al-foil placed at the backside of the nanopore membranes (see the schematic milling geometry in Figure 2). This way the smaller outlet diameter of the “conically” narrowing nanopores, which is the crucial parameter in the applications, could be assessed.

## 3. Results and Discussion—Study and Improvement of FIB Process

### 3.1. Analysis and Control of Pore Diameter Evolution

**Figure 5:**
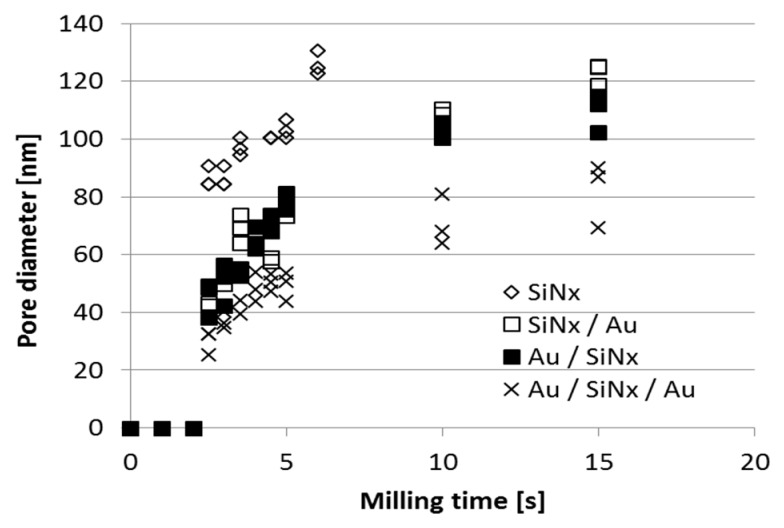
Pore diameters of different layer structures as a function of milling time by ion milling with 10pA ion current at 30keV ion energy.

The evolution of the pore diameter depends on the material composition and thickness of the dielectric membrane, as well as on the applied milling time, as plotted in Figure 5 [49]. As expected, the pore diameter evolution is faster in the thinner bare silicon-nitride membrane than in the gold covered membrane structures. In the case of shorter milling times, the zero pore diameters indicate the dose limit under which the membranes are not perforated. This beam perforation time increases with the layer thickness and decreases with the ion current.

The time or dose dependent pore diameter (*d*_p_) evolution can be described by power-laws with the distinction of two time or dose regimes. The equation was originated in the frequently applied logarithmic relationship defined between the pore diameter and the ion dose (*D*) in [50,51]: $\log(d_{p})=a+\log\left(D\right)$

(1) $$d_{p}\propto \left[\begin{array}{cc} 0 & \;\;\;\;\;\;\;\;\;\;0<t<t_{p}\\ t^{\gamma \left(t\right)} & \;\;\;\;\;\;\;\;\;\;t_{p}<t \end{array}\right]$$

where *t*_p_ was defined as perforation time indicating the transfixion event during the ion milling (when the ion beam is punching through the membrane). Parameter γ determines the pore evolution rate, which was found to change during the process. These parameters (*t*_p_ and *γ*) are complexly influenced by the ion beam properties (ion energy, ion mass, ion current) and the properties of the layer structure (sputtering rate and thickness of the different materials).

Considering the inhomogeneous cross-sectional ion intensity of the beam, Hall and Sawafta described two clearly separated regimes during He^2+^ ion beam sculpturing of silicon-nitride membranes in [31,51]. In the beginning (lower time regime), the high ion intensity in the beam centre induces an accelerated evolution of the pore diameter. Later, in the higher time regimes, the beam periphery performs the milling process with a decreased sputtering rate. For the detailed comprehension of the pore evolution rate, the defined γ parameter was calculated as the logarithm of the time dependent pore diameter functions of Figure 5:

(2) $$\gamma \left(t\right)\propto log_{t}d_{p}$$

**Figure 6:**
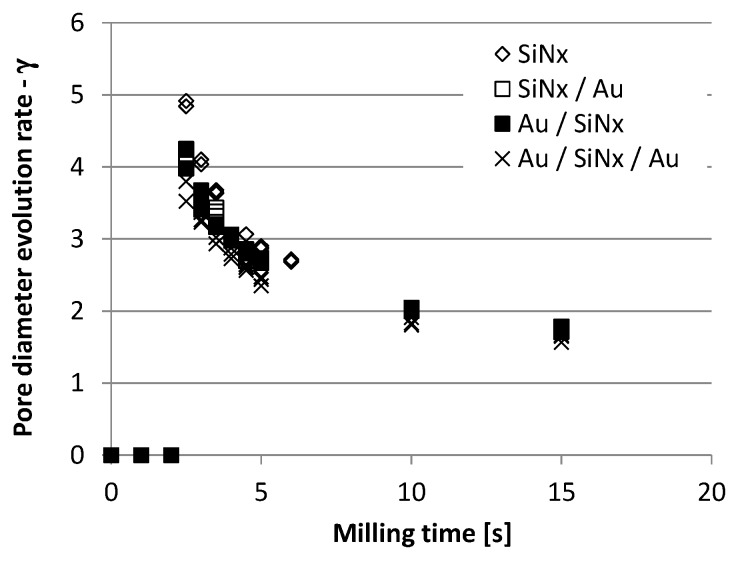
Pore diameter evolution rate parameter (*γ*) as a function of the milling time applying 10 pA ion current and 30 keV ion energy in the case of different layer structures. Values derived from the results of Figure 5.

As shown in Figure 6, in case of my assumption the γ parameter continuously decreases with milling time, contrary to Hall’s and Sawafta’s assumption. The material composition and thickness of the membrane influence the experimental *γ* parameter: in the case of the thinner silicon-nitride membrane the initial value is higher.

Therefore, a continuously decreasing pore diameter evolution rate is suggested, as the function of the milling time (or incident ion dose), instead of stationary rate regimes. The obtained function in Figure 6 can be explained by the Gaussian lateral ion intensity profile of the Ga^+^ ion beam. This assumption can account for the “saturation of the pore diameter” after a certain milling time. The final pore diameter can be defined by the geometry (diameter and convergence) and focal plane setup of the focused ion beam.

**Figure 7:**
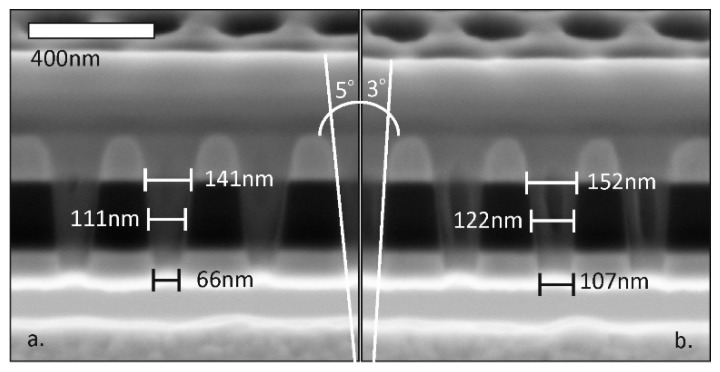
Pore geometries shaped by 5 s (**a**) and 6 s (**b**) ion milling of Au/SiN_x_/Au layer stacks when applying 5 pA Ga^+^ ion current (cross-sectional SEM view).

**Figure 8:**
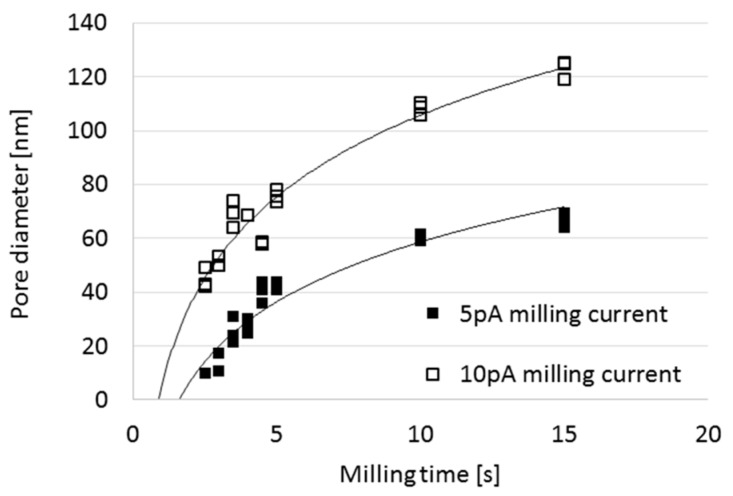
Pore diameters versus milling time in the case of Au/SiN_x_ layer structure with different ion currents (5 pA and 10 pA) at 30 keV ion energy. Note the time needed for the perforation the membrane (*t*_p_) in both cases.

The evolution of the pore shape during the milling process is demonstrated by the cross-sectional SEM views of the nanopores fabricated by different incident ion doses in Figure 7. During ion sputtering, the pore walls were approaching the surface normal, while increasing the outlet aperture of the pore. The increasing (measured) pore diameter is visualised in Figure 5 and Figure 8.

The milling current also has a significant effect on the evolving pore geometry due to the ion dose dependency of the sputtering process. This is plotted in Figure 8 for pores milled in an Au/SiN_x_ membrane from the direction of the dielectric layer. As pore diameter, the diameter of the hole obtained on the rare side of the membrane is defined. The evolution rate of the pore diameter is almost doubled comparing the cases of application 5 pA and 10 pA milling currents with the same Ga^+^ ion energy (30 keV).

**Figure 9:**
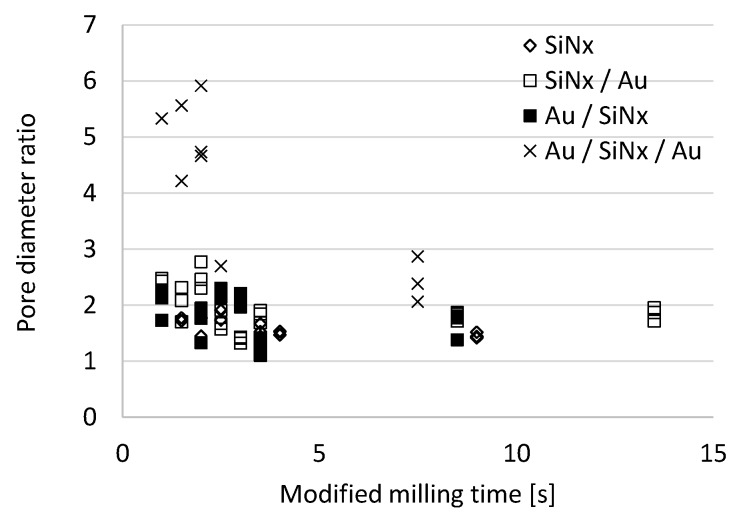
Pore diameter ratios (*d*_10pA_/*d*_5pA_) versus milling time for different layer structures and ion currents (5 pA and 10 pA, respectively). The energy of the Ga^+^ ions was 30 keV.

For a realistic comparison of the pore-shape development in the cases of drilling membranes of different thickness with changing milling conditions, a reference time has to be defined. This is the time span required to perforate the membrane (when *d*_out_ > 0), i.e., the extrapolated intersection of the pore diameter vs. milling time curve with the x-axis (*t*_p_) in Figure 8. After aligning the curves characterising different milling currents to their respective values (i.e., by shifting them parallel to the x-axis to *t*_p_ = 0), the resulting pore diameter ratios were calculated as the function of milling time elapsed after the membrane perforated and illustrated in Figure 9 in the case of various layer structures. The pore diameter ratio converges to 2 (according to the theoretical beam diameters) and the spread reduces with the milling time. Differences in the sputter-rate of the different layers (SiN_x_ and Au) will cause an additional spread in the results, certainly. The variation is larger in the case of thicker membranes in accordance with the time dependent pore diameter functions in Figure 5. Higher ratios could be experienced in the case of short drilling times due to the higher relative variation of the initial diameters of the newly perforated pores just after the beam transfixion.

Based upon this analysis of the pore formation process, the adequate ion current and milling time for the proposed pore diameter and membrane material combination can be defined. Pore diameter variation can be reduced by avoiding to set the time/current parameters close to the membrane perforation point (see Figure 8).

### 3.2. FIB Process Development

**Figure 10:**
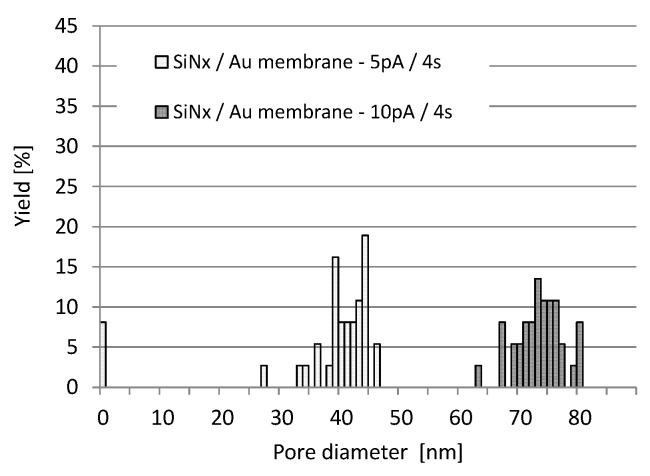
Pore diameters distribution obtained by ion milling of gold covered silicon-nitride layer structures (from the silicon-nitride side) in the case of different milling currents (5 pA and 10 pA) at 30 keV ion energy.

The statistical pore diameter distributions were defined by the practically applied process parameters and membrane structures (gold covered silicon-nitride) to improve the reproducibility of the nanopore formation process. Gold layers are adequate for the further thiol chemistry based biofunctionalisation, so hereinafter this layer structure will be discussed. The variance of the pore diameter distribution in Figure 10 is fairly large (in the range of 10 nm) for both 5 pA and 10 pA milling current, and for the lower ion current, even incomplete pore formation has to be considered [49]. The uncertainty of the milling process can be caused by charging effects on the dielectric membranes. It has to be taken into account that during Ga^+^ ion milling this electrostatic interaction causes ion beam instability by defocusing.

**Figure 11:**
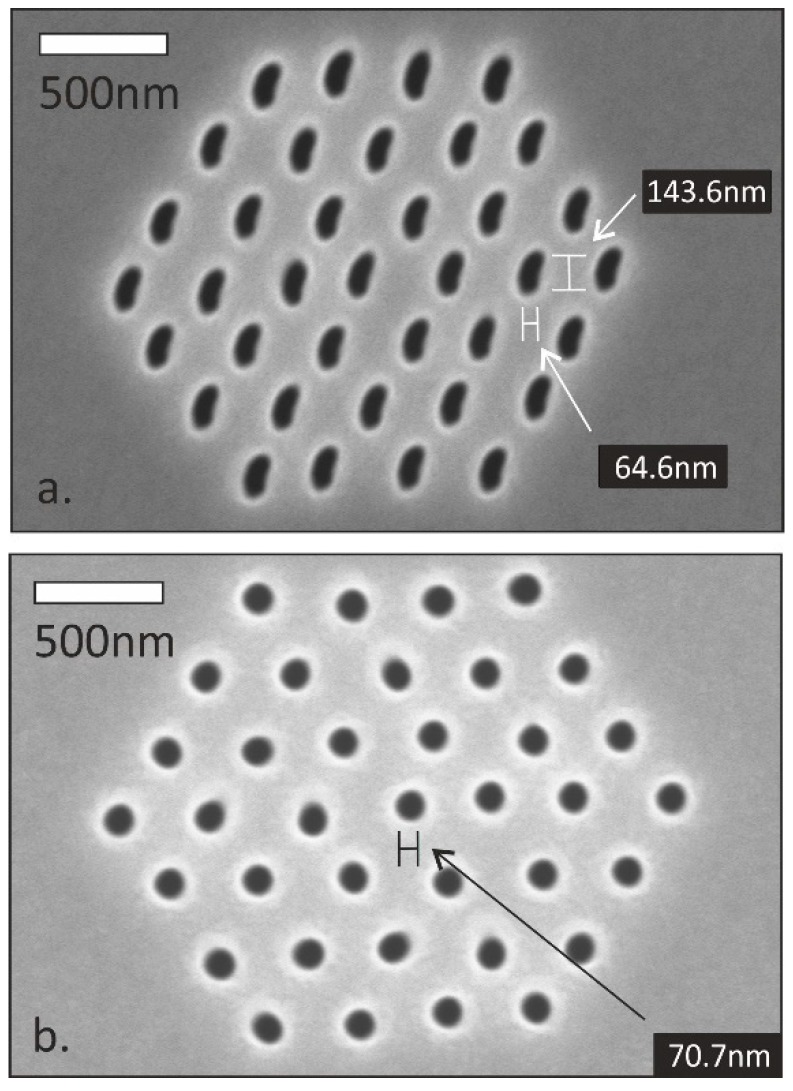
Nanopore arrays fabricated by Ga^+^ ion milling in bare (**a**) and metallised (**b**) silicon-nitride membranes. The distorted pore geometries are result of the beam defocusing effect of electrically charged dielectric membrane.

From the point of view of reproducibility, achieving a highly conformal pore size distribution with given milling conditions is a real challenge. The formed pore geometry can be significantly distorted due to the charging of the dielectric membranes and the defocusing of the incident milling Ga^+^ ion beam according to Figure 11a. An additional front size metallisation and the electrical grounding of the metallised surface improved the pore size conformity significantly (Figure 11b).

**Figure 12:**
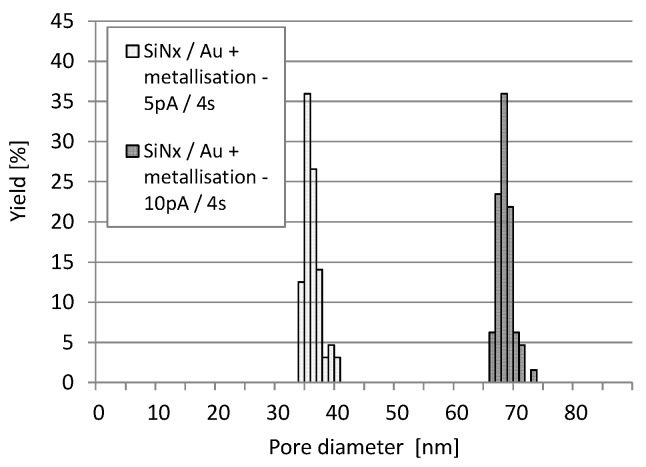
Statistical pore diameter distribution in the gold coated silicon-nitride membrane was significantly improved by grounding the additional front side metallisation (FIB parameters: 5 pA and 10 pA milling current at 30 keV for 4 s).

According to the analysis of more than 100 nanopores, discharging of the membrane layer during milling significantly reduced the spread in the pore geometry, as reflected by the narrow statistical pore size distributions in Figure 12. By this advanced technique, the pore diameter variation could be reduced to ~5 nm (5–8%). Note that the centre of the pore diameter distributions shifted to lower values as the membrane thickness increased by the additionally deposited metal layer.

### 3.3. Pore size Dependent Sensitivity of Molecule Detection

**Figure 13:**
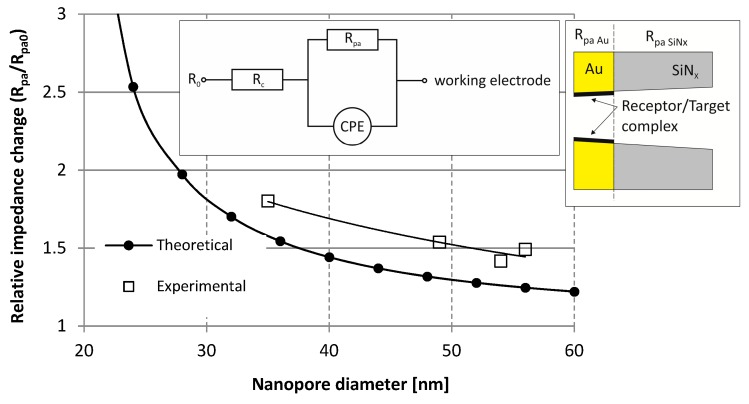
Theoretical and measured relative impedance variation of the solid-state nanopore array as a function of the nanopore-diameter after avidin binding. In the inset *R*_c_ is the resistance of the electrochemical cell, *R*_pa_ is the resistance of the nanopore array and *CPE* is the constant phase element (capacity of the membrane). *R*_pa_/*R*_pa0_ denotes the relative resistance change of the nanopore arrays, where *R*_pa0_ and *R*_pa_ are the impedance values before and after target molecule injection, respectively.

To underline the cardinal importance of the precise nanoengineering, the pore size dependent sensitivity of the impedance based molecule sensing was studied [52,53]. Gold covered silicon-nitride membranes with an array of 64 nanopores were used for monitoring avidin-biotin binding in the nanopores. The free gold surfaces were biofunctionalised by an adequate biotinylation reagent: NHS-SS-Biotin (biotin disulfide N-hydroxysuccinimide ester) utilising single step thiol chemistry [54,55]. For electrochemical analysis, 0.01 M Phosphate Buffer Saline (PBS - 0.01 M phosphate buffer, 0.0027 M potassium chloride and 0.137 M sodium chloride, pH 7.4, at 25 °C, ρ 64.8 Ω·cm) was applied as electrolyte, and PBS spiked by avidin as test sample. The avidin concentration was set to 0.1 mM to ensure the high surface coverage inside the pores. The platinum reference and working electrodes were installed on the opposite side of the nanoporous membrane. The impedance was recorded in the frequency domain of 1 Hz to 1 MHz with−0.3–0.3 V peak-to-peak potential using Gamry Reference 600 [56] electrochemical impedance spectroscope. The actual resistances of the nanopore arrays (*R*_pa_) were calculated from the results of Electrochemical Impedance Spectroscopy (EIS) using the built-in impedance models of the applied EIS300 software (Verson 6.33, Gamry Instruments, Warminster, PA, USA) (see the inset in Figure 13). Membrane resistances were measured before (*R*_pa0_) and 10, 20 and 30 min after (*R*_pa_) the sample injection and the relative change (*R*_pa_/*R*_pa0_) was calculated. The relative impedance variation values were averaged and plotted vs. nanopore diameter in Figure 13 [53].

The experimental pore array resistances were compared to the theoretical ones estimated as [11,57]:

(3) $$R_{\mathrm{pa}}=\frac{4L}{n\cdot \sigma_{s}\cdot \pi \cdot d_{\mathrm{in}}\cdot d_{\mathrm{out}}}$$

where *n* is the number, *L* is the length and din and *d*_out_ are the characteristic inlet and outlet diameters of pores, respectively. *σ*_s_ represents the specific conductivity of the electrolyte that fills the nanochannel. This theoretical consideration can estimate adequately the pore resistance as demonstrated in a preliminary work in case multi-layered nanoporous membranes, as demonstrated in [49]. To calculate the theoretical impedance variation (*R*_pa_/*R*_pa0_) of the nanopore array, homogeneous monomolecular coverage of the gold surfaces was considered in the tapering (outlet) region of the pores, thus only the outlet diameter of the pore was decreased by the molecule size. The applied physical parameters for this estimation are summarised in Table 3. [58,59,60,61]

To estimate the theoretical relative impedance change, the gold coated silicon-nitride membrane was modelled as two serially connected nanopore arrays. Contrary to the non-coated silicon-nitride layer, the pores formed in the gold layer can be easily functionalized. The resulting resistance variation is caused by the molecules having bonded in the golden pores, since the silicon-nitride layer exhibits a constant resistance (see the inset in Figure 13). As Figure 13 demonstrates, the relative pore array resistance change is significantly affected by the pore diameter in the case of a certain target molecule size. Considering the theoretical predictions, it can be assumed that the sensor signal (the relative change of membrane impedance) and thus the sensitivity of impedance based molecule sensing significantly increases as the pore diameter approaches the target molecule size.

The experimental results in Figure 13 also demonstrate a similar tendency in the case of 35 nm/49 nm/54 nm/56 nm pore diameters, however, compared to the theoretical results, higher sensor responses were detected. The theoretical calculations only considered the steric repulsion of the bound target molecule layer, although further electrochemical effects—such as electrostatic repulsion—can also appear. In case of the theoretical calculation, a monomolecular coverage was assumed, however, the formation of a self-assembled monolayer (SAM) was not proven. Note that the incomplete electrolyte filling of the nanopores or specific local ion conductivity can also significantly affect the measured resistance values. Therefore, an adequate wetting protocol is needed to make the method reliable and reproducible. [49,62]

The size dependent performance of nanopore based sensors were demonstrated and discussed in various case of applications regarding DNA detection [63], ion selectivity [64] or amino acid identification [65]. Since the resistance change is caused by pore blocking effects and so the sensitivity of the transducer is significantly influenced by the precise setting of the pore diameter to the expected size of the target molecule.

## 4. Conclusions

Considering nanopore based biosensing principles, the precise tailoring of pore geometries and adjusting to target molecule conformation and size unambiguously improves the signal-to-noise level and sensitivity of the identification method—in our case electrochemical impedance spectroscopy (EIS). Accordingly, the comprehensive pore geometry engineering is essential for the reliable and reproducible manufacturing of integrable solid state nanopore arrays for molecule diagnostic devices. Respecting the need of mechanically robust structure and area selective functionalisation of the pore surfaces, a complex material structure and a precise pore fabrication technology has to be elaborated.

In this work, the FIB milling process was considered as a flexibly applicable MEMS compatible nanostructuring method for shaping composite nanopore arrays. The evolving pore diameter depends on the material composition, and the thickness of the membrane, as well as on the applied milling time, ion dose, current and energy. The continuous evolution of pore geometries was characterized in order to define the appropriate process parameters for the reliable and reproducible manufacturing of nanopore arrays in multi-layered membranes. The resulting time (and dose) dependent pore diameter functions were determined for differently structured silicon-nitride/gold multi-layers. As a novel approach time dependent pore diameter evolution rate parameter (γ) was defined, its continuous regression during the milling process verified and interpreted by the Gaussian profile of the spatial ion intensity distribution of the ion beam. The dependency of the pore formation on the ion current was also evaluated and a linear relationship was found between the pore diameter evolution rates, excluding the vicinity of the membrane perforation occurrence. The cross-sectional nanopore geometries were also analysed by high resolution SEM, TEM and ion scanning imaging. High throughput pore diameter detection method was elaborated by detection of secondary electrons generated by translocated scanning Ga^+^ ions. Based on the comprehensive characterization adequate milling parameters (current, time) could be defined to achieve a precise and predictable pore evolution in various membrane structure. The pore diameter variation can be reduced by avoiding to choose the time/current parameters close to the membrane perforation point.

The statistical geometric parameters of a large number of nanopores were recorded and analysed. The reproducibility of the FIB nanoprocessing was improved by neutralization (electrostatic grounding) of the sample surface applying an additional conducting layer. Preventing the electrical charging of the dielectric layers can significantly decrease the resulting deterioration of the ion beam shape. By this technique the distortion of the pore diameter could be reduced significantly, and the pore size variations kept reproducibly below 5 nm.

The performance of membrane impedance analysis based molecule detection was studied by avidin-biotin binding in the nanopore array. The application of uniform pores is of pivotal importance according to theory and experiment as well, since the sensor response depends both on the concentration of the target molecule and the pore size. The development of precise nanoprocessing techniques of predictable yield is therefore an elementary requirement for molecule sensing. By this work, adequate process parameters and techniques were defined for computer-controlled FIB milling [supplementary video] of composite membranes consisting multiple nanopores with conform size. The chip level and wafer level reproducibility of nanopore formation can be an initial step towards the high throughput fabrication of solid-state nanopore based biosensor systems.

## Figures and Tables

**Table 1:** Typical techniques of fabrication solid state nanopore/nanochannel arrays (representative parameters, materials, instrumentation and process compatibility) [17,24,25,31,34,35,36,37].

Fabrication Technique		e-BEAM [17,34,35,37]	FIB or TEM [17,24,25,31,34,35,36]	NIL [34]	MEMS Based: Sacrificial Layer or Channel Sealing by Bonding [34]
**Geometry**	**Feature size**	sub 10nm	>10nm or sub 10nm	>20nm	>5nm
	**Reproducibility**	excellent	excellent	excellent	good
	**Shape**	channel array	pore/channel array	pore/channel array	lateral/buried channels
**Material**		silicon or compatible			
**Manufacturing**	**Equipment/Infrastructure**	E-BEAM system + MEMS (RIE/DRIE *)	FIB/SEM or TEM system + MEMS (RIE/DRIE *)	NIL system + MEMS (RIE/DRIE *)	MEMS
	**Cost**	High	High	High/Medium	Medium
	**Process**	Wafer scale	Chip/wafer scale	Wafer scale	Wafer scale
**Process compatibility**		MEMS			
**Main advantages**		Advanced production rate, reproducibility	Geometrical and material flexibility	Advanced production rate	Advancedproduction rate
**Main drawbacks**		High infrastructural demand			
		Complex pre- and post-processing	Moderate production rate and reproducibility	Complex pre- and post-processing	Moderate reproducibility

* RIE/DRIE: Reactive Ion Etching/Deep Reactive Iona Etching.

**Table 2:** Structural materials required for different biofunctionalisation strategies.

Proposed Surface Functionalisation	Structural Materials
thiol chemistry	Au/SiN_x_/Au SiN_x_/Au Au/SiN_x_
silane chemistry	bare SiN_x_ PFA/SiN_x_/PFA *

* perfluoroalkyl passivation.

**Table 3:** Pore and molecular parameters applied for theoretical impedance estimation [58,59,60,61].

**Pore outlet diameters**	20–60 nm
**Pore length**	450 nm (300 nm in SiN_x_ and 150 nm in Au)
**Pore wall angle (see Figure 7)**	5°
**Pore inlet diameters (estimated)**	68–108 nm
**Number of nanopores/array**	64
**Target molecule/estimated size**	Avidin/5.7 nm × 4.4 nm × 4.4 nm
**Receptor molecule/estimated length**	Biotin/2.43 nm
**Electrolyte conductivity** **(0.01 M PBS)**	150–200 S/m (15–20 mS/cm, 15–20 mho/cm)

## Supplementary Materials

The following video is available online at https://www.mdpi.com/2072-666X/10/11/774/s1, Computer controlled nanopore drilling by Focused Ion Beam (Ga^+^) milling in silicon-nitride membrane.
